# Reversible transformation and de-differentiation of human cells derived from induced pluripotent stem cell teratomas

**DOI:** 10.1007/s13577-015-0119-1

**Published:** 2015-06-12

**Authors:** Mizuna Kamada, Youji Mitsui, Taira Matsuo, Tomoko Takahashi

**Affiliations:** Laboratory of Physiological Chemistry, Faculty of Pharmaceutical Sciences at Kagawa, Tokushima Bunri University, 1314-1 Shido, Sanuki, Kagawa, 769-2193 Japan; Foundation for Advancement of International Science, Department of Research Development, Ibaraki, 305-0821 Japan

**Keywords:** hiPSC therapy, Tumor risk, Transformation, De-differentiation, Reprogramming

## Abstract

We first aimed to generate transformed cell lines from a human induced pluripotent stem cell (hiPSC)-teratoma, and then examined the tumorigenic risks of the differentiated cells from hiPSC explant, because hiPSC-derivatives give rise to tumors in immune-deficient mice when transplanted. The colonies isolated from sparse cultures of hiPSC-teratoma cells expressed *NANOG* and *OCT3/4* strongly, and telomerase reverse transcriptase (*TERT*) weakly. However, soft agar assay demonstrated that only one of them generated colonies in the gel, though hiPSCs, hTERT-transfected immortal cells, and its oncogene-transfected cells did not form any colonies. Furthermore, none of colonies isolated from the soft agar gel on primary culture (passage 0) of teratoma cells, expressed *NANOG* and *OCT3/4* in the expanded cultures. The second soft agar assay on the colony-derived cells was unexpectedly negative. The cumulative growth curve, telomere shortening, and senescence-associated β-galactosidase (SA β-gal) staining confirmed the mortality of these cells, suggesting their reversible transformation. By using medium for embryonic stem cell (ESC medium) after MCDB 131 (MCDB) medium, the differentiated culture cells derived from hiPSC-teratoma converted into the cells expressing undifferentiated marker proteins, which lost afterwords even in ESC medium with feeder SNL76/7. The reversibility of transformation and de-differentiation suggest that tumorigenic risks of differentiated cells arise when they are exposed to suitable niches in vivo. Thus, removal of only the undifferentiated cells from iPSC-derivatives before transplantation does not solve the problem. Elucidation of mechanisms of reversibility and control of epigenetic changes is discussed as a safety bottleneck for hiPSC therapy.

## Introduction

One of the criteria for pluripotency of hiPSC lines is their ability to form teratomas in immune-deficient mice [1, 2]. Thus, the safety of hiPSC lines produced by genetic introduction remains a great concern for their use in regenerative medicine [3]. Because now a pilot study to assess the safety and feasibility of the transplantation of iPSC-derived retinal pigment epithelium cell sheets in patients with exudative age-related macular degeneration has started in Japan [4], every probable tumorigenic risk should be thoroughly checked in advance.

Since tumors arise from transformed cells or remaining undifferentiated cells in iPSC-derivatives [5, 6], works to avoid the risk of tumorigenesis were focused on developments of sensitive methods to detect [7] and removal of remaining undifferentiated cells from iPSC-derivatives before their transplantation [8]. In transplants of secondary neurospheres generated from tail tip fibroblast-derived iPSCs, 84 % of mice died or became weaker because of the development of tumors. The reason was not explained well [5]. Our finding on formation of various malignant tumors due to contaminated mouse feeder cells in the explant might explain some of the reasons [9]. However, unknown mechanisms seem to exist in emergence of transformed cells from iPSC derivatives. Our recent study [10] on re-emergence of undifferentiated cells from hiPSC-derivatives prompted us to examine risks of transformation and de-differentiation of the differentiated cells. Further, a series of studies on cellular aging [11], immortalization [12], de-differentiation [10], and reprogramming [13] of human fibroblast TIG-1, urged us to isolate transformed cells from TIG-1 iPSC-teratomas and to disclose novel tumor risks of hiPSC-derivatives.

The anchorage-independent colony forming ability of cells is recognized as an indication of malignant transformation with metastatic potential [14]. Thus, we were also interested in isolating transformed cells from a hiPSC-teratoma by colony formation in a soft agar gel system.

In this paper, we aimed to generate transformed or de-differentiated cells from hiPSC-derivatives and to prove their reversibility.

The results demonstrate that removal of only the undifferentiated cells from hiPSC-derivatives before transplantation does not solve the problem. Mechanisms of the reversibility and control of epigenetic changes are discussed for prospect in hiPSC therapy.

## Materials and methods

### Cell lines and cultures

Normal human diploid fetal lung fibroblasts (TIG-1) [15], their telomerase reverse transcriptase (hTERT)-transfected immortal cell lines (IMT-1, -2, and -3) [12], their oncogene-transfected cell lines (IMT-1/RAS was generated by transfecting K-RAS12V into IMT-1, and IMT-2/BBR was established by transfecting BMI-1, BCL-2, and K-RAS12V into IMT-2), a cancer cell line (HeLa), and a feeder cell line (SNL76/7) were cultured in Dulbecco’s modified Eagle’s medium (DMEM, Invitrogen) supplemented with 10 % fetal bovine serum (FBS, Gibco). We cultured hiPSC lines (K1, K4, K12, K13, and K17) in human ESC medium with SNL76/7 feeder as described previously [13]. For culture of hiPSC-teratoma-derived cells (K4te, K12te, K13te, and K17te from respective iPSC lines) and their clones, we used MCDB 131 (Sigma) (MCDB) supplemented with 10 % FBS. We cultured soft agar-derived clones of K12te (K12te-sa clone1-4) in DMEM or MCDB containing 10 % FBS. To examine the change in the expression of undifferentiation marker proteins, we cultured K17te for 21 days in human ESC medium after MCDB medium. Population doubling level (PDL) was roughly estimated by assuming that PDL achieved 20 at cell number of 10^6^ from single cell colony.

### hiPSC generation

Plasmid DNAs of pCX-OKS-2A and pCX-c-Myc, which was used to generate mouse iPSC by Okita et al. [16], were cut with the restriction enzyme *Psh*BI. A mixtuire of equal amounts of two plasmids at mole basis was nucleofected to 1 × 10^6^ cells of TIG-1 at 17 PDL with pmax-green fluorescent protein (GFP) plasmid by using Nucleofector (Lonza) apparatus. Nucleofected cells were seeded in dishes and cultured with DMEM containing 10 % FBS. At day 7, confluent cells of each dish were trypsinized and transferred to new dishes with SNL feeder cells using human ESC medium. Valproic acid was added at the concentration of 1 mM. At day 23 and 33, ESC-like colonies were picked by ring cloning using a dissociation solution (ReproCELL, Japan). Colonies were washed with human ESC medium by centrifugation and seeded to expand, and finally stored at −150 °C using a freezing reagent (ReproCELL, Japan).

### Teratoma formation and isolation of teratoma-derived cells

We generated hiPSCs teratomas as previously described [13]. The formed tumor was fixed with 4 % paraformaldehyde solution to examine histopathology by staining with hematoxylin and eosin at Sapporo General Pathology Laboratory Co., Ltd. (Sapporo, Japan). Another part of the fresh tumor was subjected for cell isolation as described before [10]. We cultured some of the isolated cells (K12te, K17te) at an extremely low density for 2 weeks and then picked the colonies formed from single cells into dishes as clones and expanded them for further examination. K12 (passage 0) was prepared by seeding the isolated cells into dish at high density to examine anchorage independent capability at the sixth day of culture.

### RNA isolation, reverse transcription, and polymerase chain reaction (PCR)

We purified the total RNA to perform PCR with an appropriate primer set and Primestar DNA polymerase (Takara Bio, Japan) as described previously [10]. The primer sequences used are given in Table 1.

**Table 1:** Primer sequences used for RT-PCR

Gene	Direction	Sequence
*NANOG*	Forward	TGCAGTTAACATGAGTGTGGATCCAGC
	Reverse	GATCAGATCTTCACACGTCTTCAGGTTG
*h-OCT4*	Forward	GCAAGCCCTCATTTCACCAG
	Reverse	CACTCGGTTCTCGATACTGG
*h-c-MYC*	Forward	GCGTCCTGGGAAGGGAGATCCGGAGC
	Reverse	TTGAGGGGCATCGTCGCGGGAGGCTG
*h-KLF4*	Forward	ACGATCGTGGCCCCGGAAAAGGACC
	Reverse	TGATTGTAGTGCTTTCTGGCTGGGCTCC
*P53*	Forward	TGGATTGGCCAGACTGCCTT
	Reverse	CCTTCCACTCGGATAAGATGCTGA
*h-RB1*	Forward	TGGGACAGGGTTGTGTCGAA
	Reverse	TCTGAGAGCCATGCAAGGGA
*h-RAS*	Forward	TACGACCCCACTATAGAGGA
	Reverse	ACGTCATCCGAGTCCTTCAC
*P21*	Forward	TCAGAACCGGCTGGGGATGT
	Reverse	AGATGTAGAGCGGGCCTTTG
*P16*	Forward	GGGTTTTCGTGGTTCACATC
	Reverse	TTCTCAGAGCCTCTCTGGTT
*hTERT*	Forward	CCTGCTCAAGCTGACTCGACACCGTG
	Reverse	GGAAAAGCTGGCCCTGGGGTGGAGC
*CD31*	Forward	GCTGTTGGTGGAAGGAGTGC
	Reverse	GAAGTTGGCTGGAGGTGCTC
*CD34*	Forward	CTGGTCTTGGCCAACAGAAC
	Reverse	CCACGTGTTGTCTTGCTGAA
*VEGFR2*	Forward	CTGGCATGGTCTTCTGTGAAGCA
	Reverse	AATACCAGTGGATGTGATGCGG
*FSP-1*	Forward	GACAGATGAAGCTGCTTTCC
	Reverse	CATCAAGCACGTGTCTGAAG
*GAPDH*	Forward	ACCACAGTCCATGCCATCAC
	Reverse	TCCACCACCCTGTTGCTGTA
*Neo* ^*r*^	Forward	ATGGATTGCACGCAGGTTCTCC
	Reverse	TGATCGACAAGACCGGCTTCCA
*SOX2*	Forward	CTTCGCCTGATTTTCCTCGC
	Reverse	TGGGAGGAAGAGGTAACCAC
*RAF-1*	Forward	ACATGGTCCAGCTCATCGAC
	Reverse	CAGCCAATGTGGCTGTAAGG
*MDM2*	Forward	CCAGCTTCGGAACAAGAGAC
	Reverse	CAGGAAGCCAATTCTCACGA
*P14*	Forward	CCAACGGAGTCAACCGTTTC
	Reverse	TTGGAGTGAACGCATCGACT
*BCL2*	Forward	TGGCCTTCTTTGAGTTCGGT
	Reverse	TCACTTGTGGCCCAGATAGG
*BMI1*	Forward	AGCAGAAATGCATCGAACAA
	Reverse	CCTAACCAGATGAAGTTGCTGA
*REX1*	Forward	CAGATCCTAAACAGCTCGCAGAAT
	Reverse	GCGTACGCAAATTAAAGTCCAGA

### Isolation of anchorage-independent growing cell colonies and their cultures

We used trypsin treatment to harvest the cells for the soft agar assay. In the case of hiPSCs, we added 5 μM the Rho-associated protein kinase (ROCK) inhibitor, thiazovivin (Stemgent), to the cell suspension following the protocol of the manufacture. We seeded 1.0 × 10^5^ to 1.0 × 10^6^ cells in 5–8 mL of a 0.33 % upper agar layer on 1.0 % under agar layer with DMEM containing 10 % FBS. To prevent the gel from drying, we added an appropriate medium for each culture to the gel with 1 week interval. After culturing for 2–4 weeks, we counted the colonies consisting of more than 100 actively growing cells in the gel as the number of anchorage-independent colonies. For further experiments, we picked up the colonies using a tip and expanded them in separate dishes for culture.

### Analysis of telomere length

We isolated genomic DNA from culture cells and tumor tissues to examine telomere lengths using the TeloTAGGG telomere length assay kit (Roche, Switzerland) and determined the mean length of telomere restriction fragment (TRF) using Telometric 1.2 as described before [12].

### Detection of senescence-associated β-galactosidase activity

The growth-arrested cells were fixed, washed, and incubated with staining solution as described previously [12]. We detected the stained blue cells for senescence-associated β-galactosidase (SA β-Gal) activity and counted the percentage of the stained cells.

### Immunocytochemistry and quantification of undifferentiation marker proteins

We performed immunocytochemistry of OCT4, NANOG, and SSEA4 to quantify the fluorescent area for analysis of significant difference. Their primary antibodies were detected by Alexa594, Alexa488, and Alexa555, respectively. Slides were observed with Axiovert 200 M fluorescence microscope system (Carl Zeiss, Germany) after staining. Details are described previously [10].

## Results and discussion

### Isolation of clonal colonies from the hiPSC-teratoma and their transformed characteristics

**Fig. 1:**
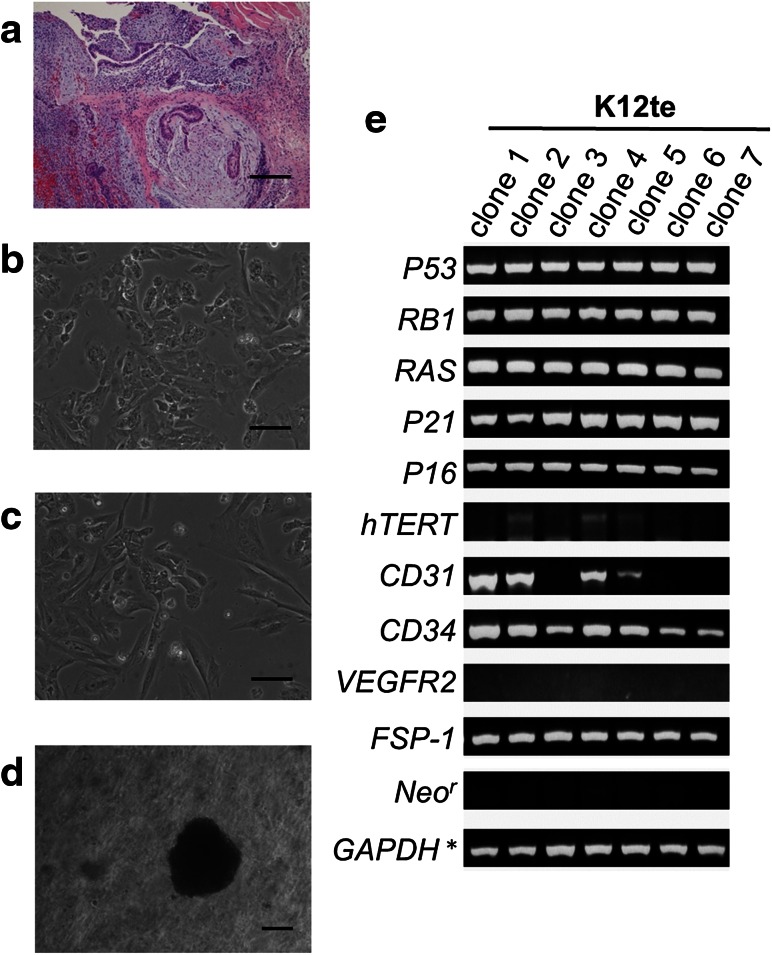
Isolation of cloned cells from hiPSC-teratoma, gene expression, and transformation. **a** Histopathology of K12 teratoma. **b** Clone 2 and **c** clone 4 are colonies from the teratoma. **e** Gene expression analyses of growth regulating genes and differentiation genes. **d** Colonies in the soft agar gel were formed only from clone 4. Clone 1, 2, 3, 4, 5, 6, and 7 in this paper corresponds to L2, L4, L9, L11, L12, R4, and R7, respectively in our previous paper [10], from which expression of GAPDH* was referred. The *bars* indicate 100 μm (**a**, **b**, **c**) or 200 μm (**d**)

Among 22 hiPSC lines (K1–K22) [13] generated from TIG-1, K12 and K17 hiPSC lines were selected to generate teratoma in a severe combined immunodeficient mouse. Their histopathological analysis demonstrated the presence of glandular epithelium, connective tissue, and blood vessels in K12 teratoma (Fig. 1a) and of glandular epithelium, cartilage like tissue, and blood vessels in K17 teratoma (Fig. 4a). First, we isolated cells from K12 hiPSC-teratoma (K12te) and cultured them at a very low density for single cell colonies. We isolated rapidly growing colonies with different morphologies (Fig. 1b, c) from the culture and expanded them in separate dishes for further analysis of gene expression. We found that any clones did not express the neomycin resistant gene (*Neo*^*r*^) (Fig. 1e), confirming that the cells were derived from hiPSCs but not mouse feeder cells, as aforementioned [9]. The fibroblast secretary protein-1 (*FSP*-*1*) gene was expressed in all the clones, suggesting that they were not epithelial cells. Among endothelial cell marker genes, *CD31* (*PECOM*-*1*) was expressed in clones 1, 2, 4, and 5, and vascular endothelial growth factor receptor 2 (*VEGFR*-*2*) was not expressed in any clones. However, *CD34*, a marker of the progenitors of vascular cells, was expressed in all clones. Thus, it seemed that these cells were progenitors of the vascular tube at different stages of differentiation. The analyses also showed that cell cycle regulatory genes or tumor suppressor genes (*RB*, *P53*, *P21*, and *P16*) were expressed in all the clones. Among reprogramming genes, *NANOG* was expressed in clones 1, 2, and 4; *OCT3*/*4* in clones 2, 4, and 5; and *c*-*MYC* and *KLF4* in every clone as shown previously [10].

Then, we selected clone 2 (Fig. 1b) and clone 4 (Fig. 1c) to isolate transformed cell lines, because they expressed four reprogramming genes and *hTERT*. The soft agar assay demonstrated that only clone 4 generated approximately 100 colonies in the gel (one of the single colonies is shown in Fig. 1d). And, clone 2 did not generate any colonies (K12te-clone 2 in Table 2), even though they expressed reprogramming genes (strongly) and *hTERT* (slightly) like undifferentiated hiPSCs did. This suggests that the remaining undifferentiated cells are not necessarily transformed cells. Because many large colonies were formed in the gel from clone 4, we isolated colonies into culture for analysis of transformed nature. However, the isolated cells lost their growth capability after 10–20 PDLs, suggesting a reversible nature of their transformation.

**Table 2:** Colony formation of human cell lines in a soft agar gel

Characteristics	Transgenes	Names of cell lines	Colony formation in soft agar gel
Normal cell	No	TIG-1	nd^a^
Cancer cell	No	HeLa	+
Immortal cell	*hTERT*	IMT-1	−
		IMT-2	−
		IMT-3	−
Oncogene-transfected cell	*hTERT, K-RAS* ^*12V*^	IMT-1/RAS	−
	*hTERT, BMI-1, BCL-2, K-RAS* ^*12V*^	IMT-2/BBR	−
hiPSC	*Oct4, Sox2, Klf4, c-Myc*	K1	−
		K4	−
		K12	−
Teratoma-derived cell	*Oct4, Sox2, Klf4, c-Myc*	K4te (15 PDL)	−
		K13te (4 PDL)	−
		K12te clone 2 (26 PDL)	−
		K12te clone 4 (27 PDL)	+^b^
		K12te (Passage 0)	+^c^
Cell from colony^c^	*Oct4, Sox2, Klf4, c-Myc*	K12te-sa clone 1 (26 PDL)	−
		K12te-sa clone 1 (57 PDL)	−

Anchorage-independent growth capability was positive in K12te at passage 0 (c) and K12te clone 4 at 27 PDL (b). Note that the colony formation of K12te-sa clone 1 (26 and 57 PDL) was negative after expansion from the first gel colony of K12te (passage 0)

*PDL* population doubling level

**Fig. 2:**
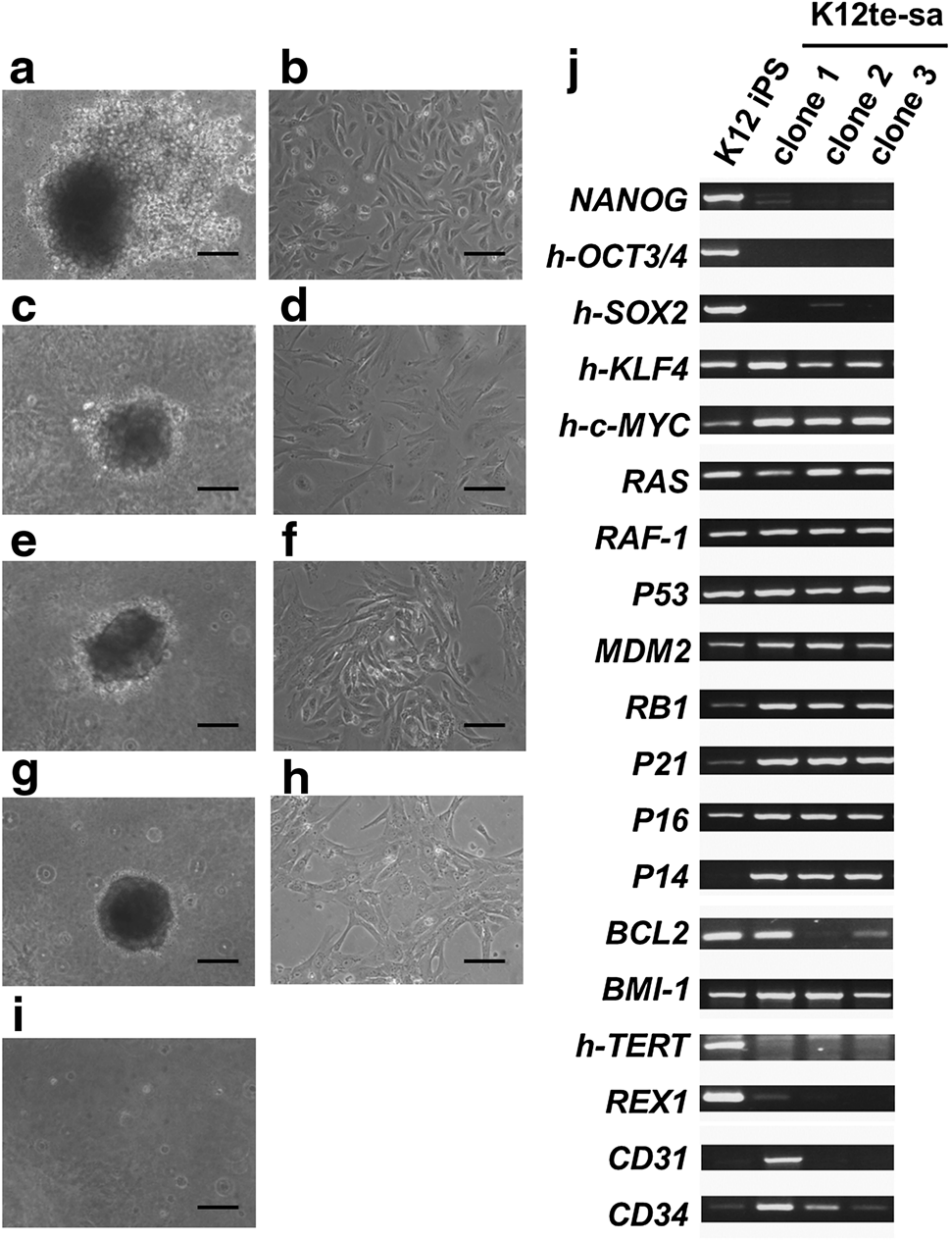
Generation of colonies using primary cells from hiPSC-teratoma and reversible transformation. The soft agar colony (**a**, **c**, **e**, and **g**) and their isolated culture cells (**b**, **d**, **f**, and **h**) are shown by phase- contrast microscopy; K12te-sa clone 1 (**a**, **b**), clone 2 (**c**, **d**), clone 3 (**e**, **f**), and clone 4 (**g**, **h**), respectively. **i** K12 hiPSC did not generate any colonies. **j** Note that any cloned cell line did not express the undifferentiated genes. The *bars* indicate 100 μm

^a^ *nd* not determined, ^b^ shown in Fig. 1, ^c^ shown in Fig. 2

### Transformed cells from a primary culture of hiPSC-teratoma and their reversible nature

Because rapidly growing colony cells at an extremely low-density culture exhibited a transient nature of transformation irrespective of their expression of undifferentiated cell markers, we questioned if transit transformation happened during sub-cultivation. Therefore, we checked existence of transformed cells in primary cells (passage 0) of K12te. Soft agar assay of the cells (K12te passage 0 in Table 2) demonstrated formation of 18 big colonies at 4 weeks. We picked up colonies into separate dishes for further culture and established 8 clones (K12te-sa clones 1–8). Four colonies (clones 1, 2, 3, and 4) in the gel (Fig. 2a, c, e, g, respectively) showed some differences in the morphology (Fig. 2b, d, f, h, respectively). Gene expression analysis of three clones (clones 1, 2, and 3) demonstrated that they did not express reprogramming genes (*NANOG*, *OCT3/4*, and *SOX2*) in contrast to their high expression in the original K12 iPSC (Fig. 2j). Furthermore, undifferentiated cell marker genes, *REX1* and *hTERT*, were not expressed in these clones. These findings indicate that transformed cells exist in a teratoma, though they are not derived from remaining undifferentiated cells.

### Anchorage-independent growth capability of various human cell lines in a soft agar gel

We considered that soft agar gel colony formation of iPSC-teratoma-derived cells was an indication of the generation of malignantly transformed cells, since anchorage-independent cell growth signature identifies tumors with metastatic potential [14]. However, the present findings that the transformed cells from hiPSC-teratoma are not remaining undifferentiated cells, and lost their growth capability after isolation into culture, prompted us to re-evaluate anchorage-independent growth capability by using various human cell lines that we have established from the same parent cell line, TIG-1. A positive control of human cancer cell line, HeLa, formed colonies (over 1000) in gel as expected. As shown in Table 2, K12 hiPSC-teratoma-derived cells (K12te) at passage 0, and K12te-clone 4 (at 27 PDL), generated colonies, though K4te at 15 PDL and K13te at 4 PDL were negative. Furthermore, immortal cell lines (IMT-1, -2, and -3) and oncogene-transfected cell lines (IMT-1/RAS and IMT-2/BBR) did not form any colonies. Thus, the colony-forming ability in a soft agar gel shown here present resemblance to malignant transformation rather than just immortalization as reported [14]. In addition, it is noteworthy that hiPSC lines (K1, K4, and K12) did not generate any colonies even in the presence of a ROCK inhibitor (used to promote survival of isolated single hiPSC). This is in accordance with a report that a soft agar colony formation assay was unable to detect hiPSCs in the presence of a ROCK inhibitor [7]. Furthermore, it is notable that colony formations of the second assay were unexpectedly negative on K12te-sa clone 1 (at 26 and 57 PDL) when subjected for the assay, after expansion of the soft agar colony derived from K12te (passage 0). Thus, it is noteworthy that their transformed property is not robust but reversible.

### Gene expression and replicative senescence of transformed cells

We expanded three clones (K12te-sa clones 1, 2, and 3) from the transformed cells with anchorage-independent growth ability (K12te passage 0) for gene expression analysis comparing with parent K12 hiPSC (Fig. 2j). They expressed oncogenes (*RAS*, *RAF*-*1*, *MDM2*, and *BMI1*) and anti-oncogenes (*RB1*, *P53*, *P21*, *P16*, and *P14*) at similar levels. Expression of *BCL2*, an oncogene with anti-apoptotic action, was high in K12te-sa clone 1, but low in the other 2 clones. A marker of endothelial cells, *CD31* and a marker of its progenitor, *CD34*, were highly expressed only in clone 1.

**Fig. 3:**
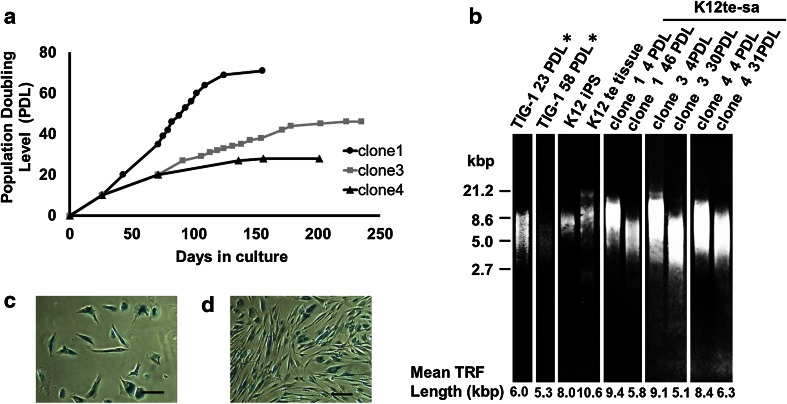
Cumulative growth curve, telomere length, and SA β-Gal staining. **a** K12te-sa clone 1 (*filled circle*), clone 3 (*filled square*), and clone 4 (*filled traingle*) lost their growth capability. **b** Telomere restriction fragment (TRF) was elongated in hiPSC and the teratoma tissue, from TRF in parent cell line, TIG-1, and it decreased at late passages in each clone. Percentage of SA β-Gal staining in clone 1 (**c**) and clone 3 (**d**) was about 95 %. Thus, replicative senescence of colony-derived cells from the soft agar gel was confirmed. *TRF of TIG-1 was referred from our previous paper [12]

Because we found the second soft agar assay of clone 1 was negative (K12te-sa clone 1 in Table 2) as mentioned above section, irrespective of positive result in the first assay (K12te passage 0 in Table 2), we performed a long-term subcultivation of clones 1, 3, and 4 to determine if they were mortal or immortal. A cumulative growth curve (Fig. 3a) demonstrates that all of them were mortal (clone 1 ceased to grow at 71 PDL, clone 3 at 46 PDL, and clone 4 at 28 PDL). Then, we analyzed changes in the telomere length during their subcultivation (Fig. 3b). The average TRF length in K12 hiPSCs and K12 teratoma were 8.0 and 10.6 kbp, respectively. It is noteworthy that the reprogrammed cells and the teratoma cells had longer telomeres than did parent young TIG-1 cells (6.0 kbp). In addition, it is apparent that the telomeres of each clone at 4 PDL became shortened at their late passages (K12te-sa clone 1, from 9.4 to 5.8 at 46 PDL; clone 3, from 9.1 to 5.1 at 30 PDL; and clone 4, from 8.4 to 6.3 at 31 PDL in Fig. 3b) indicating their proliferative senescence. Next, we examined SA β-Gal staining at the terminal stage of cell culture. Their senescence was confirmed by 94.7 % blue cell staining in clone 1 and 96.2 % in clone 3 (Fig. 3c, d, respectively). Loss of anchorage-independent growth capability during expansion culture would be due to proliferative senescence, though a possibility of terminal differentiation may not be excluded. Thus, we confirmed a reversible nature of the transformation of these cells.

### Clonal differentiated cells from hiPSC-teratoma and the reversible nature of de-differentiation

**Fig. 4:**
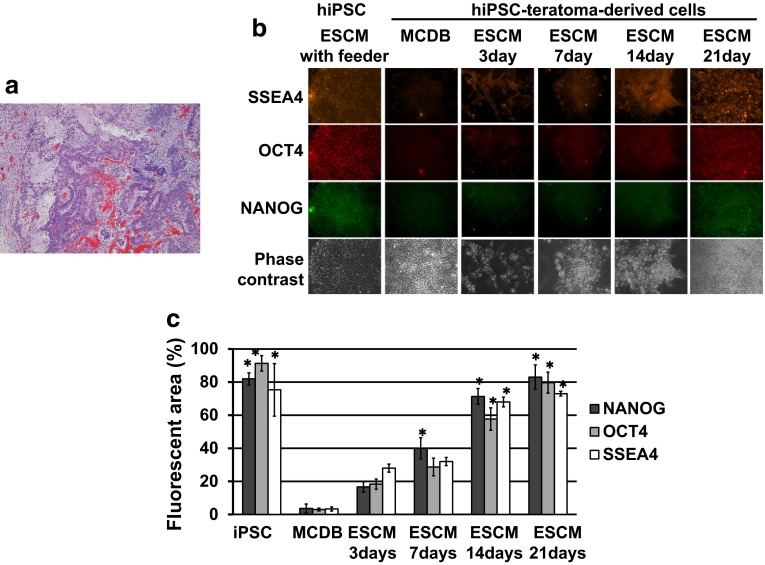
Histopathology of the K17 hiPSC-teratoma and conversion of hiPSC-teratoma-derived differentiated cells to the cells with undifferentiation marker proteins. **a** The K17 hiPSC line formed a teratoma with glandular epithelium, cartilage-like tissue, and vascular tube. **b** Immuno-cytochemical detection of undifferentiated cell marker proteins. Clone of K17te was cultured with MCDB medium (MCDB) or ESC medium (ESCM) Phase-contrast images are shown in panels of Phase-contrast. **c** The mean percentages of fluorescent area of NANOG, OCT4, and SSEA4, were determined by analyzing three photographs for each sample. *P < 0.05, statistical significance from the value at MCDB

Because we knew a reversible nature of the transformation (Figs. 1, 2, 3), we speculated that similar reversibility might occur in the differentiation of cells from the teratoma. Thus, we performed extremely low density-cultures of K17hiPSC-teratoma-derived cells (K17te) (Fig. 4a for histopathological staining) with MCDB medium and obtained a several growing colonies in dishes. We isolated and expanded them as clones revealing epithelial-like morphology (Fig. 4b, panel of MCDB and Phase-contrast). After seeding them in five 3-cm dishes, we changed the medium from MCDB to ESC, which has been previously used for proper hiPSC culture. However, ESC medium causes damage to differentiated cells. Immunocytochemical analyses demonstrated that the undifferentiated cell marker proteins, SSEA4, OCT4, and NANOG did not exist during the culture in MCDB medium, but appeared at the third day after medium change to ESC medium and increased in their cell numbers with successive days of culture (Fig. 4b). We observed damages and loss of the differentiated cells under phase-contrast microscopy. Because the culture was not supported by a feeder cell layer, the remaining undifferentiated cells, if any, would not self-replicate. Thus, an overwhelming number of cells with undifferentiation marker proteins which increased in number after medium change, must be de-differentiated cells which bear resemblance to hiPSC-like cells. Quantitative analyses on areas of SSEA4, OCT4, and NANOG staining showed that quantities of each marker proteins of de-differentiated cells reached to the level of hiPSC (Fig. 4c). Thus, we assume that differentiated cells converted into de-differentiated cells by unknown factors during culture at a severe condition with ESC medium. However, their morphological features were not similar to those of true hiPSCs. Then, we isolated the de-differentiated cell colonies to culture with a feeder layer in ESC medium which have been used for proliferation of hiPSCs. All of our trials to obtain stable hiPSC-like lines have failed, suggesting the transit conversion of de-differentiated state. Thus, we confirmed the reversible nature of both differentiation and de-differentiation.

### New insights into tumorigenic risks and possible mechanisms of reversibility in transformation and de-differentiation

Reversibility of de-differenation and transformation was proved in the teratoma cells from hiPSC lines generated with inserted genes. It leads us to new insights into tumorigenic risks of hiPSC derivatives transplanted. First, removal of only the undifferentiated cells from iPSC derivatives before transplantation, as focused in previous works [7, 8], does not solve the problem.

Second, it is required to perform extensive studies on unsolved questions to elucidate molecular mechanisms of the reversibility.

The hiPSCs in this paper were generated by introduction of CAG promoter-drive OSK2A and CAG promoter-drive c-Myc plasmids as shown [16]. Thus, a possible expression of these exogenous genes might cause the reversibility in the cells. However, we should test the hypothesis of the reversibility using hiPSC lines generated without viral vectors [16] or by direct introduction of reprogramming proteins [17], which have no inserted genes, and also using embryonic stem cell (ESC) lines generated from inner cell mass.

Each line of ESC or iPSC holds some donor cell characteristics and thereby they are not identical in ability to differentiate and in safety for cell therapy. Furthermore, derivatives of even germ-line-competent iPSCs lines allowed the generation of residual undifferentiated cells in adult mice [18]. This suggests that undifferentiated iPSC-like cells could not be eliminated by extended cell differentiation.

Process of iPSC generation is stepwise resetting of epigenetic landscape as reported [19]. Thus, we suppose that chromatin modifications in iPSCc have become more susceptive to environmental changes. The most important chromatin modification is methylation or de-methylation of DNA and histones as well as levels of acetylation and citrullination of histones, which might be involved in reversibility of transformation and de-differentiation in hiPSC-derivative cells.

As for unknown factors in ESC medium to convert cells into de-differentiated cells, constituents in ESC medium such as FGF-2 and 2-mercapto-ethanol as well as chemical agents known to alter chromatin conformations are worth testing their effects on emergence of transformed cells or de-differentiated cells.

Finally, we should look beyond just improving the reprogramming process [20] or removing only remaining undifferentiated cells in advance [3]. The epigenomes of both pluripotent cells and differentiated cells are not robust as expected [19] but flexible with changes of cellular environments.

Results of further extensive studies on new mechanisms should be used to remove tumorigenic risks for regenerative therapy with hiPSCs.
